# Renal complications in chronic hypoparathyroidism – a systematic cross-sectional assessment

**DOI:** 10.3389/fendo.2023.1244647

**Published:** 2023-11-02

**Authors:** Karen Gronemeyer, Carmina Teresa Fuss, Franca Hermes, Armin Plass, Ann-Cathrin Koschker, Anke Hannemann, Henry Völzke, Stefanie Hahner

**Affiliations:** ^1^Department of Medicine I, Division of Endocrinology and Diabetes, University Hospital, University of Wuerzburg, Wuerzburg, Germany; ^2^Institute of Clinical Chemistry and Laboratory Medicine, University Medicine Greifswald, Greifswald, Germany; ^3^German Centre for Cardiovascular Research, Partner Site Greifswald, Greifswald, Germany; ^4^Institute for Community Medicine, University Medicine Greifswald, Greifswald, Germany

**Keywords:** Hypoparathyroidism, renal calcification, Renal Insufficiency, ultrasound, Parathyreoidectomy

## Abstract

**Context:**

Although renal long-term complications are acknowledged in chronic hypoparathyroidism (HPT), standardized investigations are scarce.

**Objective:**

To systematically investigate renal complications and their predictors in hypoparathyroid patients compared to matched individuals.

**Design:**

Prospective observational study in 161 patients with chronic HPT.

**Methods:**

Patients received renal ultrasound, clinical and laboratory assessments. An individual 1:3 matching with participants from the German population-based Study of Health in Pomerania was performed.

**Results:**

Of 161 patients (92% postoperative HPT), prevalence of eGFR <60ml/min/1.73m^2^ was 21%, hypercalciuria 41%. Compared to healthy individuals, HPT patients had a significantly lower eGFR (74.2 vs. 95.7 ml/min/1.73m², p<0.01). Renal ultrasound revealed calcifications in 10% (nephrocalcinosis in 7% and calculi in 3%). Patients with renal calcifications had higher levels of 24-hour urine calcium excretion (8.34 vs. 5.08 mmol/d, p=0.02), spot urine calcium excretion (4.57 vs. 2.01 mmol/L, p=0.01) and urine calcium-to-creatinine ratio (0.25 vs. 0.16, p<0.01) than patients without calcifications. Albumin-corrected calcium, phosphate, calcium-phosphate product, 25-hydroxyvitamin D in serum, eGFR, daily calcium intake or disease duration were not significantly different between these two groups. Including patients receiving rhPTH therapy, a lower serum phosphate concentration (odds ratio 1.364 [95% confidence interval (CI) 1.049-1.776], p<0.05) and a longer disease duration of HPT (odds ratio 1.063 [95% CI 1.021-1.106], p<0.01) were significant predictors for renal calcifications. Excluding patients receiving rhPTH therapy, a higher 24-hour urine calcium excretion (odds ratio 1.215 [95% CI 1.058-1.396], p<0.01) was a significant predictor for renal calcifications but not serum magnesium or disease duration.

**Conclusions:**

Prevalence of impaired renal function among patients with chronic HPT is increased and independent from visible renal calcifications. Depending on exclusion of patients with rhPTH therapy, regression analysis revealed disease duration and serum phosphate or disease duration and 24-hour urinary calcium excretion as predictors for renal calcifications.

**Clin Trials Identifier:**

NCT05585593

## Introduction

Chronic hypoparathyroidism (HPT) is a rare disease characterized by low circulating calcium concentrations due to inappropriate parathyroid hormone (PTH) secretion. The most common cause of HPT is damage, accidental removal or devascularization of the parathyroid glands (1). Current standard therapy consists of supplementation of calcium and active vitamin D (2). For patients suffering from insufficiently controlled HPT or complications as renal insufficiency or calcifications a replacement therapy with recombinant human (rh) PTH is available (3, 4). Despite these treatment options, patients, even those with well-controlled HPT, suffer from comorbidities and long-term complications such as cataracts, psychiatric diseases or an impaired quality of life (5–8). Renal insufficiency defined as an estimated glomerular filtration rate (eGFR) <60 ml/min/1.73m² has been reported in 12% to 41% of HPT patients, whereas renal calcifications occurred in 11% to 43% (9–18). So far, only a small number of prospective or cross-sectional studies with a maximum of 130 patients with postsurgical HPT systematically assessed renal function and performed renal ultrasound (5, 10, 12, 19).

Physiologically, 98% of filtered calcium is reabsorbed in the kidneys, whereas phosphate is reabsorbed up to 2.5 mg per 100 ml glomerular filtrate (20). The reabsorption of both calcium and phosphate as well as magnesium is regulated by PTH, the calcium sensing receptor (CaSR), fibroblast growth factor 23 (FGF23) and sodium (20). PTH binding to the PTH1 receptor in the kidney results in an increased calcium reabsorption in the cortical thick ascending limb and distal convoluted tubule and a decreased phosphate reabsorption in the proximal tubules. Therefore, the absence or inadequate response to PTH in HPT patients leads to an increased calcium excretion and phosphate reabsorption resulting in hypocalcaemia und hyperphosphatemia (21–23).

The predictors and risk factors leading to renal complications in treated HPT are still unclear. A reduction in renal function was correlated with higher age, longer disease duration, hypercalcemia and an elevated calcium-phosphate-product (9, 11, 24, 25). Hypercalcemia, hyperphosphatemia and a higher calcium-phosphate-product have been reported as predictors for nephrocalcinosis (1, 14, 24). The influence of 24-hour calcium excretion on renal calcifications is still unclear as previous studies showed discordant results (12, 13, 17).

In this study, we aimed to further investigate renal comorbidities, especially renal insufficiency and calcifications, in HPT patients. Therefore, we compared a well-characterized cohort of HPT patients to matched individuals from the general German population. Furthermore, we intended to identify risk factors leading to renal insufficiency or renal calcifications.

## Methods

### Patients with hypoparathyroidism

**Figure 1:**
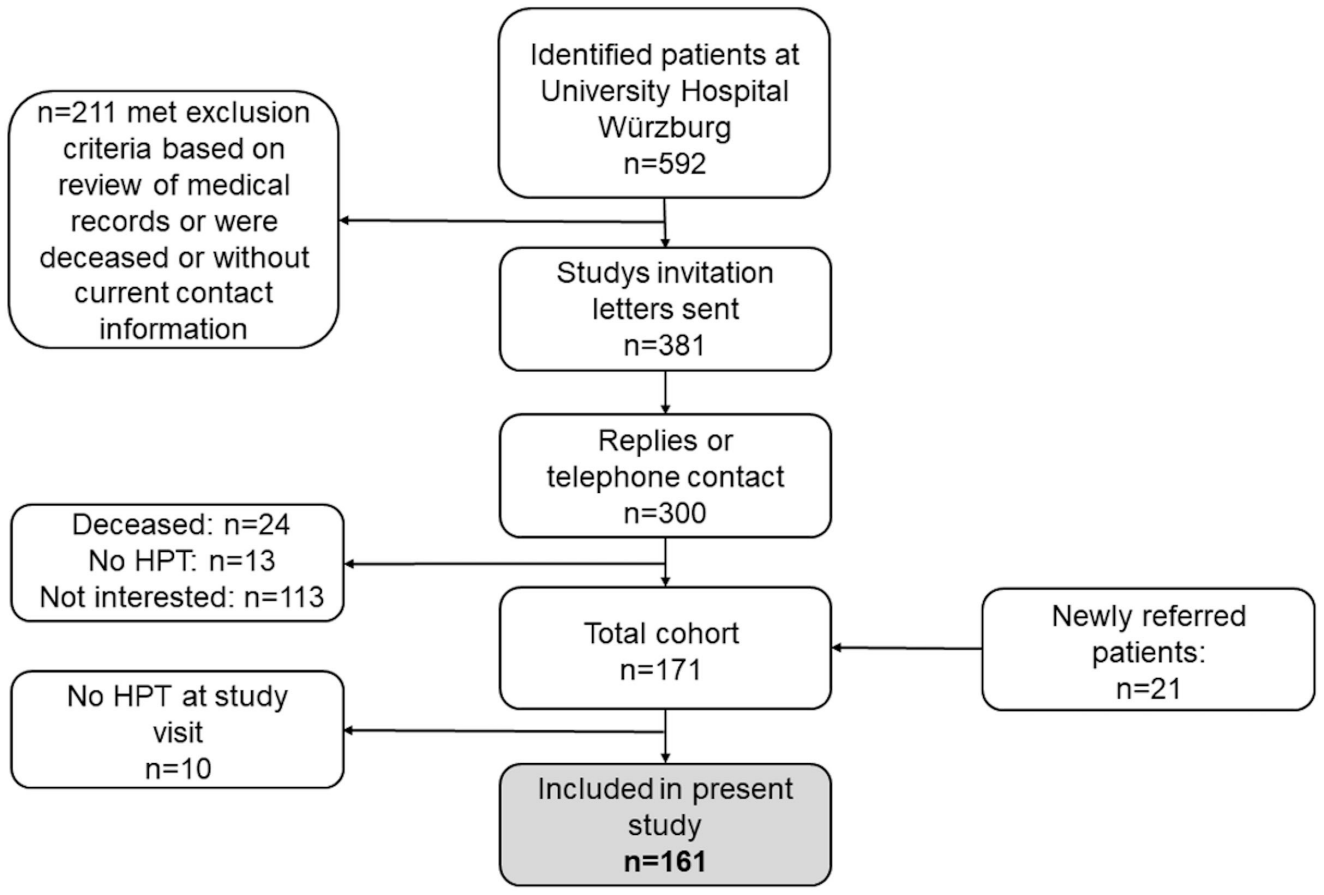
Flow chart of recruitment process for the present study. HPT, Hypoparathyroidism.

Among 592 patients treated at the University Hospital Wuerzburg between 2014 and 2017 with the diagnosis “hypoparathyroidism” in their medical reports, 381 met the inclusion criteria for the present study. Inclusion criteria were age ≥18 years, written informed consent and established diagnosis of hypoparathyroidism for at least 6 months. The diagnosis required at least one documented hypocalcaemia in combination with an inappropriately low PTH level. Furthermore, 21 newly referred patients were included in the present study. The recruitment process for the present study is shown in **Figure 1**. The final study population consisted of 161 HPT patients enrolled between 2017 and 2019. At the day of study visit, patients were instructed to skip breakfast and not to take their daily HPT specific medication. Medication, medical history and symptoms of hypocalcaemia were documented by using a standardized questionnaire. Medication was classified according to the anatomical therapeutic chemical (ATC) classification (26). The study was approved by the Ethics Committee of the University of Wuerzburg (No. 13/17).

### Laboratory parameters

Venous blood samples were drawn and analyzed in the endocrine service laboratory or the central laboratory of the University Hospital Wuerzburg. Patients collected a 24-hour urine prior to the study visit. Serum concentrations of calcium, phosphate, magnesium, albumin, lipids, creatinine as well as spot urine calcium and 24-hour urine calcium excretion were measured using an automated biochemical analyzer (Cobas Integra 800 and Cobas C701, Roche Diagnostics, Mannheim, Germany). An IDMS standardized enzymatic assay (Cobas CREP2 Creatinine plus ver.2 (System-ID 2046 001)) was used for creatinine analysis. Albumin-corrected serum calcium was calculated according to Payne et al. (27). The eGFR was calculated using the Modification of Diet in Renal Disease equation (28, 29). For comparison with SHIP individuals, the eGFR was calculated using the Chronic Kidney Disease Epidemiology Collaboration (CKD-EPI) equation (30). Serum TSH was measured using the chemiluminescent immunoassay IMMULITE 2000 Third Generation TSH System Analyzer (Siemens Healthcare Diagnostic, Eschborn, Germany). Serum intact PTH was measured using the chemiluminescent enzyme immunoassay IMMULITE 2000 Systems Analyzer (Siemens Healthcare Diagnostic, Eschborn, Germany), whereas serum 25-OH vitamin D was analyzed using the automated chemiluminescent immunoassay IDS-iSYS 25 VitD assay (Immuno Diagnostic Systems, Boldon, UK). Hypercalciuria was defined as urine calcium excretion ≥7.5 mmol/24h for men and ≥6.25 mmol/24h for women according to the Endocrine Society Guidelines (31). Albuminuria was defined as urine albumin-creatinine ratio (UACR) ≥30 mg/g, microalbuminuria as UACR 30-300 mg/g and macroalbuminuria as UACR >300mg/g (32). Hypocalcemia was defined as serum albumin – corrected calcium <2.0 mmol/L.

### Renal ultrasound and resting blood pressure measurement

Renal ultrasound was performed using Siemens Acuson S2000 (Siemens Medical Solutions, Erlangen, Germany) in order to assess renal calcifications (33). An independent, experienced internist reviewed all ultrasound images and videos and classified them as “positive” or “negative” for nephrolithiasis or nephrocalcinosis.

After a 5-minute resting period in sitting position, systolic and diastolic blood pressures were measured three times using boso – medicus uno blood pressure monitor (Boso medicus, Jungingen, Germany). The mean value of the second and third measurement was used for statistical analyses.

### Healthy individuals

Data were obtained from the Study of Health in Pomerania-TREND (SHIP-TREND) cohort, a population-based study conducted in West Pomerania, Germany. Details on study design, sampling methods and examination protocols have been reported previously (34, 35). All participants underwent standardized medical examinations, including blood sampling and an extensive computer-aided personal interview on health-related lifestyle and medical histories. Systolic and diastolic blood pressures were measured three times on the right arm of seated subjects, using HEM-705CP (Omron Corporation, Tokyo, Japan). Mean systolic and diastolic blood pressure was calculated using the second and third measurement. Medication data was classified using the ATC classification. Fasting was requested, but not mandatory. Venous blood samples were taken in the mornings between 7 a.m. and 1 p.m., stored at -80°C in the Integrated Research Biobank of the University Medicine Greifswald and used in accordance with its regulations (36). Serum concentrations of electrolytes, lipids and TSH were measured using the automated biochemical analyzer Dimension Vista (Siemens Healthcare Diagnostic, Eschborn, Germany). Creatinine concentrations were measured using an IDMS standardized enzymatic assay on the Dimension Vista (Siemens Healthcare Diagnostic, Eschborn, Germany). The eGFR was calculated using the CKD-EPI equation. Albuminuria was defined as in HPT patients. All participants provided written informed consent. The study conformed to the principles of the Declaration of Helsinki and was approved by the Ethics Committee of the University of Greifswald.

### Statistical analysis

Characteristics of HPT patients and individuals from the general German population were expressed as median with 1^st^-3^rd^ quartile for continuous variables or as frequency with proportion for categorical variables. For the comparison of patients with and without renal calcifications, all those receiving rhPTH replacement therapy (n=9) were excluded. RhPTH replacement therapy is restricted to a small group of selected patients, which may introduce a potential bias. Group differences between HPT patients with and without renal calcifications were analyzed using Mann–Whitney *U* test (continuous variables) or Fisher’s exact test (categorical variables).

The association of renal calcifications and clinical and biochemical characteristics was analyzed using binary logistic regression with backward selection. Independent variables for regression analysis were 24-hour urine calcium excretion (mmol/d), serum phosphate (mmol/dL), serum magnesium (mmol/dL), serum calcium-phosphate-product (mmol^2^/dL^2^) and disease duration (years). 24-hour urine calcium excretion was significantly different between patients with and without renal calcifications. Serum phosphate (24), calcium-phosphate-product (14, 16) and magnesium (37–39) as well as disease duration (16, 40) were considered as predictors for renal calcifications in recently published studies. Spot urine calcium and urine calcium-creatinine-ratio (UCCR) were not included in the logistic regression model due to significant correlations with 24-hour urine calcium excretion.

**Figure 2:**
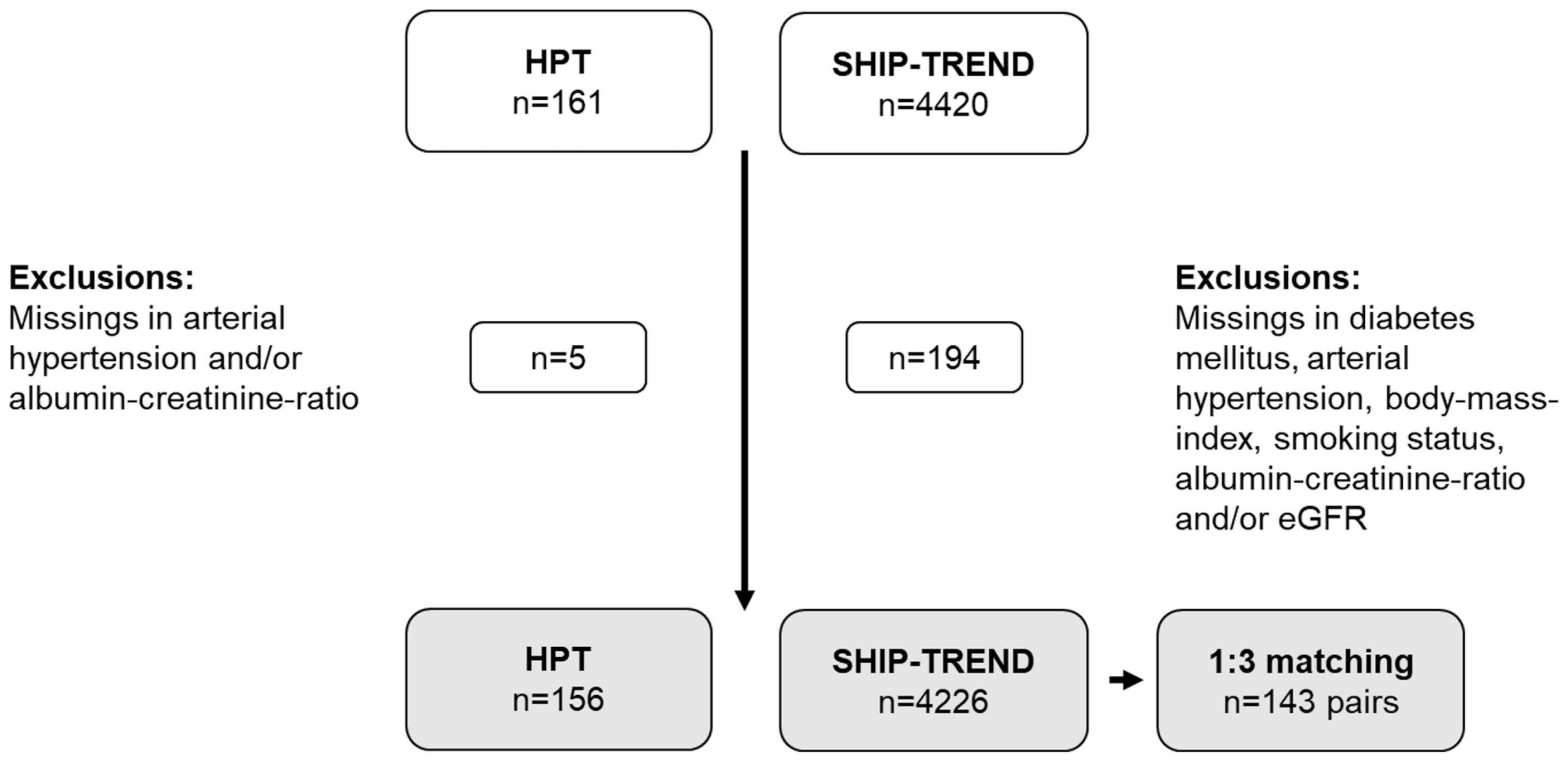
Selection of the study population for matching of HPT patients and SHIP-TREND individuals. Matching variables used individual 1:3 matching were age group, sex, BMI, diabetes mellitus, arterial hypertension and smoking status. HPT, Hypoparathyroidism.

To assess a possible relation between HPT and renal function we compared HPT patients with healthy individuals. We used an individual 1:3 matching to control confounding factors (**Figure 2**).The matching was performed using the greedy matching algorithm implemented in a SAS macro (41). The matching was based on age group (20-39 years, 40-49 years, 50-59 years and ≥60 years), sex (male/female), BMI (± 3 kg/m²), self-reported current smoking (yes/no), self-reported diabetes mellitus (yes/no) and hypertension (yes/no). Hypertension was defined as systolic blood pressure ≥140 mmHg or diastolic blood pressure ≥90 mmHg or intake of antihypertensive medication (ATC C02, C03, C07, C08, C09 with the exclusion of sole intake of thiazides). The 1:3 matching identified 150 patients and 450 control individuals who were further analyzed. Group differences between HPT patients and individuals were tested for statistical significance with Friedman’s Chi-Square test (continuous and ordinal variables) or Cochran-Mantel-Haenszel-test (dichotomous variables).

A p-value <0.05 was considered statistically significant. All statistical analyses were performed with SPSS version 28.01 or SAS 9.4 (SAS Institute Inc., Cary, North Carolina, USA).

## Results

### General characteristics of patients with hypoparathyroidism

Baseline characteristics of 161 HPT patients included in the present study are given in **Table 1**. Regarding treatment of HPT, most patients were taking oral calcium supplements in combination with active vitamin D (46.4%), 21.7% were additionally taking cholecalciferol. Only nine patients (5.6%) were treated with PTH, among whom four received teriparatide and five rhPTH (1–84). Six of these patients were additionally treated with oral calcium supplements and/or active vitamin D and/or vitamin D3. Only two patients were taking neither oral calcium supplements nor active vitamin D, PTH or vitamin D3. 15.6% of HPT patients presented with low magnesium levels, out of whom 16% already supplemented magnesium.

**Table 1:** General characteristics of patients with hypoparathyroidism.

Characteristics	HPT patients n=161
Age, years	56.0 (48.0 – 64.0)
Sex, female, n (%)	123 (76.4)
BMI, kg/m²	26.7 (23.9 – 32.8)
Disease duration, years	13 (5 – 22)
Postsurgical HPT, n (%)	148 (91.9)
Oral calcium supplements, n (%) Daily dose, mg/d	127 (78.9) 1000 (500 – 1200)
Active vitamin D, n (%)	137 (85.1)
Alfacalcidol, n (%) Daily dose, mg/d	4 (2.5) 1.25 (1.00 – 1.88)
Calcitriol, n (%) * Daily dose, µg/d	115 (71.4) 0.50 (0.50 – 0.75)
Dihydrotachysterol, n (%) * Daily dose, mg/d	19 (11.8) 0.5 (0.5 – 1.0)
Vitamin D3, n (%) Daily dose, I.E./d	52 (32.3) 1600 (800 – 2900)
Magnesium, n (%) Daily dose, mg/d	25 (15.5) 300.0 (90.0 – 487.5)
rhPTH, n (%)	9 (5.6)
Teriparatide rhPTH (1–34), n (%) Daily dose, µg/d	4 (2.5) 19.5 (18.3 – 27.1)
rhPTH (1–84), n (%) Daily dose, µg/d	5 (3.1) 75.0 (55.0 – 100.0)
Diuretics, n (%) Daily dose, mg/d	43 (26.7) 12.5 (12.5 – 25.0)
Thiazides, n (%)	35 (21.7)
Loop diuretics, n (%)	7 (4.3)
Potassium-sparing diuretics, n (%)	1 (0.6)
Serum total calcium, mmol/L†	2.10 (2.00 – 2.30)
Serum albumin-corrected calcium, mmol/L†	2.00 (1.90 - 2.10)
Serum phosphate, mmol/L†	1.33 (1.18 – 1.47)
Serum 25-OH vitamin D, µg/L	31.25 (25.70 – 40.00)
Serum magnesium, mmol/L†	0.78 (0.72 – 0.82)
Urinary creatinine, mg/24 hours	1329.0 (1119.9 – 1775.5)
Urinary calcium, mmol/24 hours	5.33 (2.70 – 8.70)
Hypercalciuria, n (%)	64 (40.5)
eGFR, ml/min/1,73m²	71.0 (62.0 – 82.0)
eGFR <60 ml/min/1,73m², n (%)	34 (21.1)

*1 subject taking calcitriol and dihydrotachysterol was included in both categories.

† Normal values: serum total calcium 2.0 – 2.7 mmol/L, serum albumin-corrected calcium 2.0 – 2.7 mmol/L, serum phosphate 0.87 – 1.45 mmol/L, serum magnesium 0.70 – 1.05 mmol/L.

BMI, body mass index; eGFR, estimated glomerular filtration rate; HPT, hypoparathyroidism: rhPTH, recombinant human parathyroid hormone.

Data are given as median (1st – 3rd quartile) or frequency (proportions). BMI, body mass index; eGFR, estimated glomerular filtration rate; HPT, hypoparathyroidism: rhPTH, recombinant human parathyroid hormone.

### Renal insufficiency in HPT

**Figure 3:**
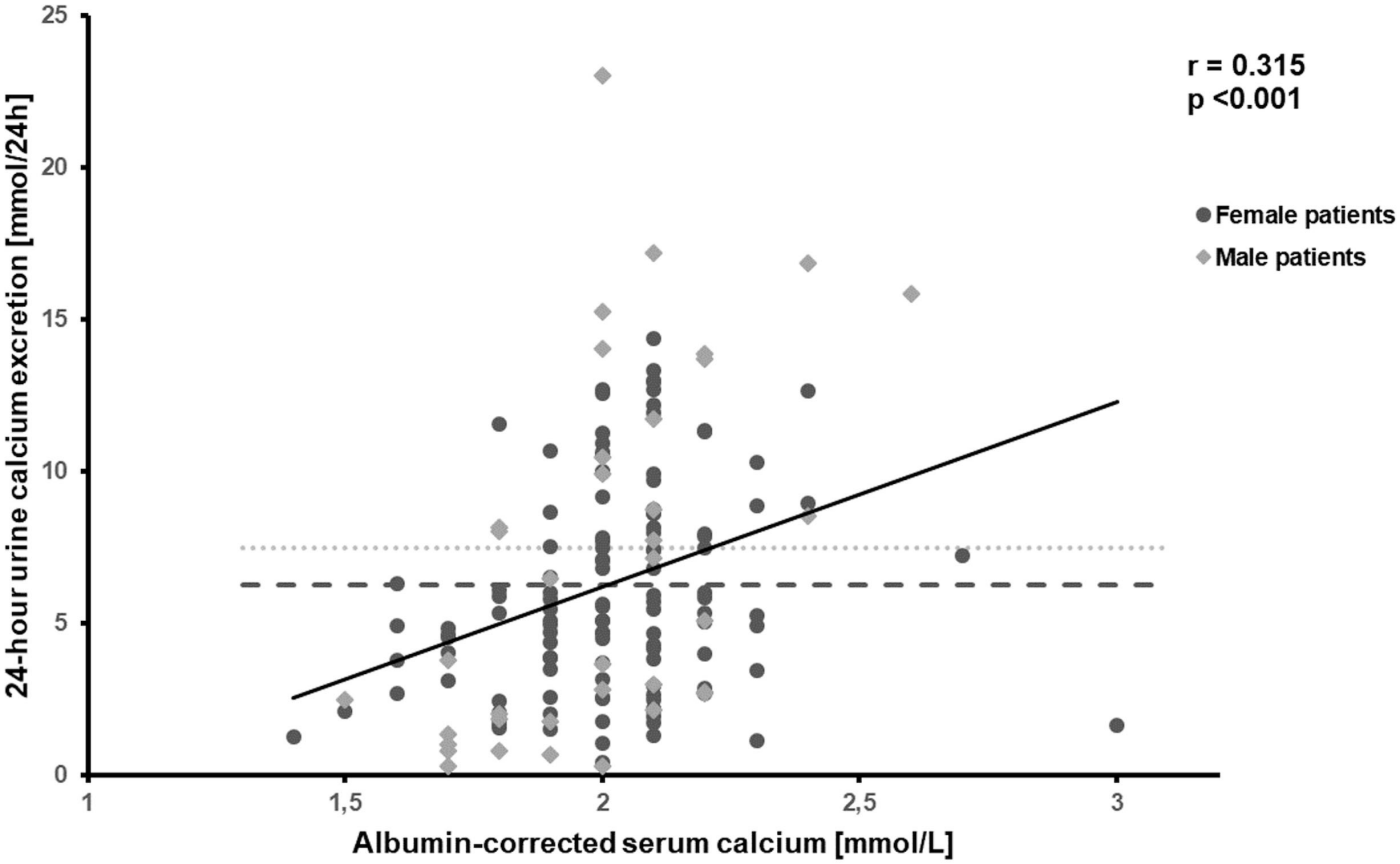
Correlation between albumin-corrected serum calcium and 24-hour urine calcium excretion, r = -0.315, p<0.001. Hypercalciuria in male (>7.5 mmol/24h, 43%) and female patients (>6.25 mmol/24h, 40%). ULN=Upper limit of normal.

An eGFR below 60 ml/min/1.73m² was found in 21% of HPT patients. 43% of male and 39% of female patients suffered from hypercalciuria (**Figure 3**). 28.1% of hypercalciuric HPT patients already received thiazides, however, 51.4% of patients already receiving thiazide treatment were still hypercalciuric. Significant positive correlation was observed between serum creatinine and age (r = 0,247, p<0.01) and inverse correlation between eGFR and duration of HPT (r = -0.283, p<0.001) as well as eGFR and serum albumin-corrected calcium (r = -0.168, p<0.05). Furthermore, 24-hour calcium excretion was positively correlated with serum albumin-corrected calcium (r = 0.315, p<0.001) (**Figure 3**), but not with daily calcium dose. In contrast, there was no correlation between eGFR and serum phosphate, calcium-phosphate product or UCCR.

**Figure 4:**
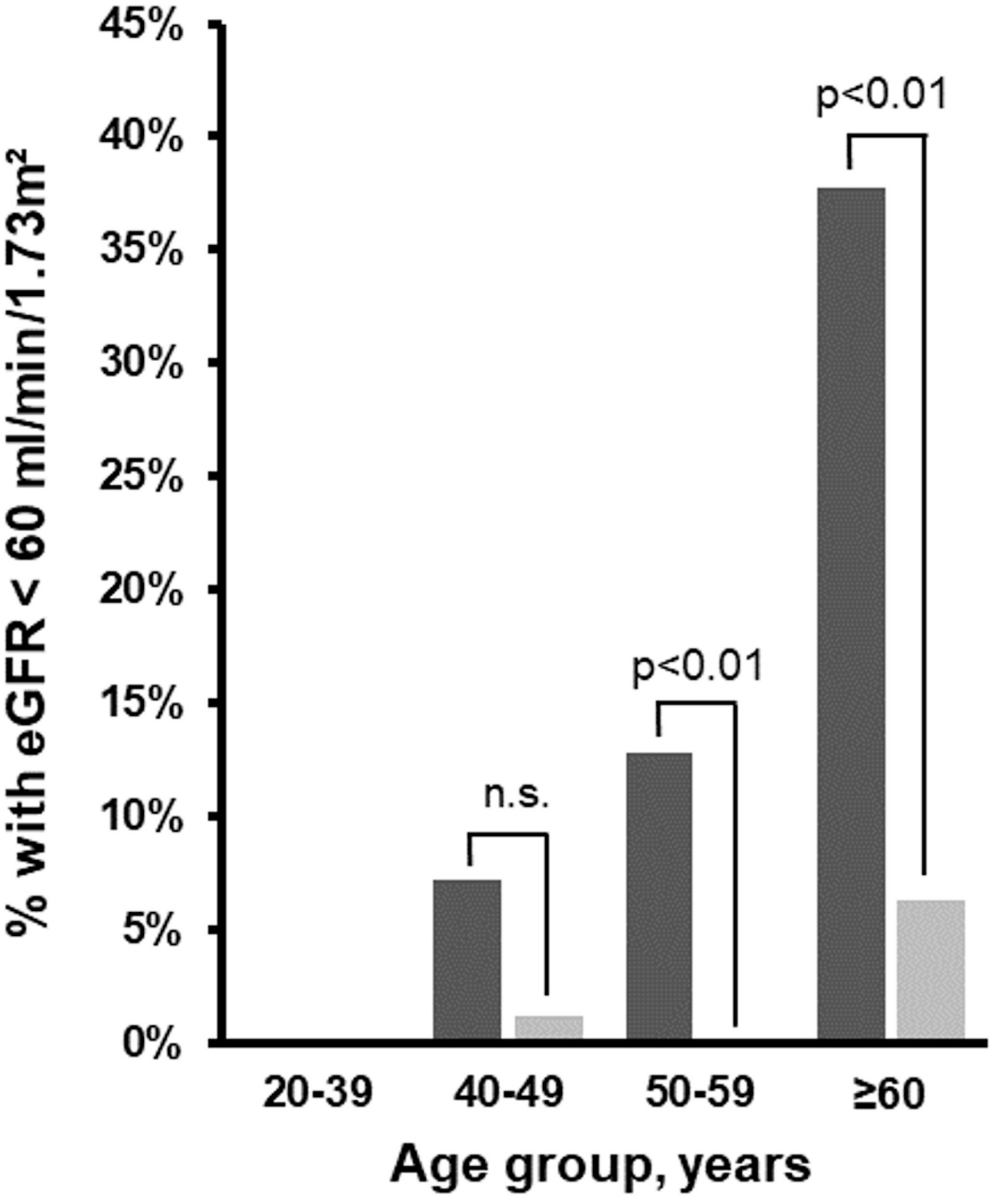
Proportion of HPT patients (n=143) and matched SHIP-TREND individuals (n=429) with eGFR<60ml/min/1.73m² stratified by age group. *Black columns*, HPT cohort; *grey columns*, SHIP individuals. eGFR, estimated glomerular filtration rate; HPT, hypoparathyroidism.

In comparison to matched individuals, HPT patients had a significantly lower eGFR. Analyses stratified by age group further demonstrated higher proportions of renal insufficiency in HPT patients aged between 40 to 49 years, 50 to 59 years and 60 years or older (**Figure 4**). However, HPT patients demonstrated significantly lower UACR than matched individuals while the proportion of albuminuria was comparable between the groups. Furthermore, HPT patients had significantly lower serum calcium and magnesium concentrations and higher serum phosphate concentrations (**Table 2**). These differences persisted also after comparison of HPT patients to healthy individuals additionally matched for thiazide intake (**Tables S1**, **S2**).

**Table 2:** Characteristics of HPT patients and healthy individuals after 1:3 matching for age group (20-39, 40-49, 50-59, ≥60 years), sex, BMI ( ± 3 kg/m²), current smoking, diabetes mellitus and history of hypertension.

Characteristics	HPT patients	SHIP-TREND n=429	p-value
	n=143		
Female, %	74.8	74.8	–
Age, years	56.0 (48.0 – 64.0)	56.0 (47.0 – 65.0)	0.38
BMI, kg/m²	26.6 (23.6 – 30.5)	26.5 (23.7 – 30.6)	0.76
Current smoking, %	14.7	14.7	–
Diabetes mellitus, %	2.80	2.80	–
TSH, mU/L	0.70 (0.10 – 1.80)	1.17 (0.72 – 1.59)	<0.01
Serum total calcium, mmol/L†	2.10 (2.00 - 2.30)	2.29 (2.23 - 2.34)	<0.01
Serum albumin-corrected calcium, mmol/L†	2.02 (1.92 – 2.12)	2.31 (2.24 – 2.37)	<0.01
Serum magnesium, mmol/L†	0.78 (0.73 – 0.82)	0.85 (0.80 – 0.91)	<0.01
Serum phosphate, mmol/L†,**	1.33 (1.18 – 1.45)	0.99 (0.88 – 1.10)	<0.01
Systolic BP, mmHg*	127 (118 – 138)	127 (116 – 140)	0.82
Diastolic BP, mmHg*	81.5 (75.0 – 90.0)	77.0 (70.5 – 83.5)	<0.01
Hypertension, %	59.4	59.4	–
Intake of			
any antihypertensive medication, %	51.8	46.4	0.10
diuretics, %	25.2	6.06	<0.01
betablockers, %	24.5	29.1	0.21
calcium channel blockers, %	14.0	6.06	<0.01
RAAS Inhibitor, %	36.4	33.1	0.35
other antihypertensives, %	2.80	0.70	0.05
eGFR, ml/min/1.73m²	74.2 (64.4 – 84.5)	95.7 (84.4 – 103.5)	<0.01
Urine albumin-to-creatinine, mg/g	8.90 (6.13 – 11.90)	11.1 (6.60 – 19.3)	<0.01
Albuminuria, %			0.10
None, %	93.0	88.1	
Microalbuminuria, %	5.59	10.7	
Macroalbuminuria, %	1.40	1.17	

*1 missing value in HPT patients and SHIP-TREND participants, respectively.

**1 missing values in SHIP-TREND participants.

† Normal values: serum total calcium 2.0 – 2.7 mmol/L, serum albumin-corrected calcium 2.0 – 2.7 mmol/L, serum phosphate 0.87 – 1.45 mmol/L, serum magnesium 0.70 – 1.05 mmol/L.

Data are median (1st – 3rd quartile) or proportions. Group differences were tested with Friedman’s Chi-Square test (continuous and ordinal variables) or Cochran-Mantel-Haenszel test (dichotomous variables). Microalbuminuria was defined as urine albumin-to-creatinine ratio 30-300 mg/g, macroalbuminuria was defined as urine albumin-to-creatinine ratio >300 mg/g. BMI, body mass index; BP, blood pressure; eGFR, estimated glomerular filtration rate; HPT, hypoparathyroidism; RAAS, renin-angiotensin-aldosterone system; TSH; thyroid stimulating hormone.

### Renal calcifications in HPT

Renal ultrasound was performed in 150 HPT patients (93.1%) revealing nephrolithiasis in 3.3% and nephrocalcinosis in 7.3%. In total, renal calcifications, defined as nephrolithiasis and/or nephrocalcinosis, were diagnosed in 10.0% of HPT patients (one patient had both nephrolithiasis and nephrocalcinosis).

**Figure 5:**
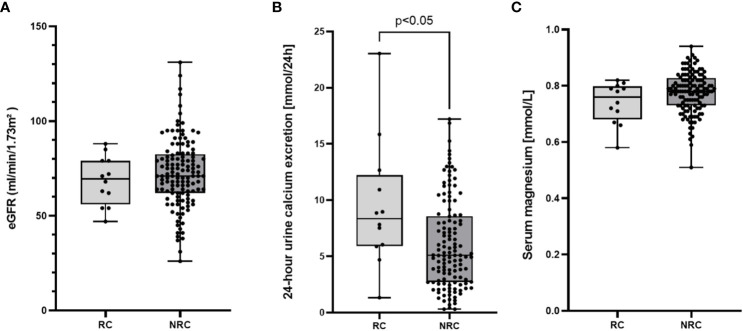
Box plots with individual data points illustrating estimated glomerular filtration rate (eGFR, A), 24-h urine calcium excretion **(B)** and serum magnesium concentration **(C)** in patients with (RC, n=12) or without (NRC, n=129) renal calcifications.

In the following, we compared all patients with renal calcifications to those without after excluding subjects with rhPTH therapy. This allowed us to identify factors influencing the occurrence of renal calcifications (**Table 3**). The two groups were comparable regarding etiology of HPT, disease duration, serum electrolytes and eGFR. Furthermore, intake of oral calcium supplements, active vitamin D, cholecalciferol and magnesium was comparable. However, 24-hour urine calcium excretion, spot urine calcium excretion, UCCR and daily dose of diuretics differed significantly between patients with and without renal calcifications (**Figure 5**).

**Table 3:** Characteristics of patients with or without renal calcifications (defined as nephrocalcinosis or nephrolithiasis). AI using rhPTH therapy were excluded.

Characteristics	Renal Calcifications n=12	No Renal Calcifications n=129	p-value
Female, n (%)	10 (83.3)	100 (77.5)	1.00
Age, years	53.0 (35.8 – 64.8)	56.0 (48.0 – 64.0)	0.53
Postsurgical HPT, n (%)	11 (91.7)	119 (92.5)	1.00
Disease duration, years	15.0 (5.0 – 30.3)	12.0 (5.0 – 22.0)	0.43
Serum total calcium, mmol/L**†**	2.20 (2.03 – 2.45)	2.10 (2.00 – 2.30)	0.33
Serum albumin – corrected calcium, mmol/L**†**	2.05 (1.93 – 2.38)	2.00 (1.90 – 2.10)	0.20
Serum phosphate, mmol/L**†**	1.25 (1.01 – 1.45)	1.35 (1.23 – 1.49)	0.21
Serum magnesium, mmol/L**†**	0.76 (0.68 – 0.80)	0.78 (0.73 – 0.83)	0.13
Serum 25 – OH vitamin D, µg/L	32.1 (25.9 – 42.7)	31.0 (24.6 – 40.5)	0.60
Serum calcium – phosphate – product, mmol²/l²	2.69 (2.45 – 3.10)	2.90 (2.58 – 3.16)	0.32
Urinary calcium, mmol/24 hours	8.34 (5.91 – 12.23)	5.08 (2.70 – 8.55)	0.02
Spot urine calcium, mmol/L	4.57 (2.20 – 5.37)	2.01 (0.95 – 3.28)	0.01
Urine calcium – to – creatinine ratio	0.25 (0.21 – 0.31)	0.16 (0.08 – 0.25)	<0.01
Hypercalciuria, n (%)**	8 (66.7)	48 (37.8)	0.07
eGFR, ml/min/1.73m²	69.5 (56.0 – 79.0)	71.0 (62.0 – 82.5)	0.45
eGFR **<**60 ml/min/1.73m², n (%)	3 (25.0)	28 (21.7)	0.73
Oral calcium supplements, n (%)	8 (66.7)	106 (82.2)	0.24
Daily dose, mg/d	900 (600 – 1000)	1000 (500 – 1213)	0.74
Active vitamin D, n (%)††	11 (91.7)	112 (86.8)	1.00
Alfacalcidol daily dose, µg/d	1.5 (1.5 – 1.5)	1.0 (1.0 – 1.0)	1.00
Calcitriol daily dose, µg/d	0.50 (0.43 – 0.75)	0.50 (0.50 – 0.75)	0.53
Dihydrotachysterol daily dose, µg/d	0.5 (0.5 – 0.5)	1.0 (0.5 – 1.0)	1.00
Vitamin D3, n (%)	2 (16.7)	41 (31.8)	0.35
Oral magnesium supplements, n (%)	1 (8.3)	16 (12.4)	1.00
Diuretics, n (%)	4 (33.3)	32 (24.8)	0.50
Daily dose, mg/d	25.0 (25.0 – 43.8)	12.5 (12.5 – 25.0)	0.01

*1 missing in subjects without renal calcification

**2 missings in subjects without renal calcification.

† Normal values: serum total calcium 2.0 – 2.7 mmol/L, serum albumin-corrected calcium 2.0 – 2.7 mmol/L, serum phosphate 0.87 – 1.45 mmol/L, serum magnesium 0.70 – 1.05 mmol/L.

†† There were no significant differences between the two groups in the intake of alfacalcidol, calcitriol and dihydrotachysterol.

BMI, body mass index; eGFR, estimated glomerular filtration rate; rhPTH, recombinant human parathyroid hormone.

Patients using rhPTH therapy were excluded. Data are given as median (1st – 3rd quartile) or frequency (proportions). Group differences were tested with Mann–Whitney U test (continuous variables) or Fisher’s exact test (categorical variables). BMI, body mass index; eGFR, estimated glomerular filtration rate; rhPTH, recombinant human parathyroid hormone.

To identify factors predicting the probability of renal calcifications, we performed logistic regression analysis. Including patients receiving rhPTH therapy, a lower serum phosphate concentration (odds ratio 1.364 [95% confidence interval (CI) 1.049-1.776], p<0.05) and a longer disease duration of HPT (odds ratio 1.063 [95% CI 1.021-1.106], p<0.01) were significant predictors for renal calcifications, whereas 24-hour urinary calcium excretion was elevated in patients with renal calcifications but failed to reach statistical significance (odds ratio 1.137 95% CI 0.999-1.294, p=0.0053]) (**Table S3**). Excluding patients receiving rhPTH therapy, a higher 24-hour urine calcium excretion (odds ratio 1.215 [95% CI 1.058-1.396], p<0.01) was a significant predictor for renal calcifications but not serum magnesium or disease duration (**Table S4**).

## Discussion

Including 161 HPT patients, our study belongs to the largest investigations on renal function and calcifications among HPT patients. Furthermore, our study is based on real-life data regarding management and comorbidities of HPT patients. Other prospective studies investigating renal comorbidities among HPT patients were limited to a maximum of 130 HPT patients (10, 12, 19) or restricted to non-surgical HPT (14). By performing renal ultrasound in 93% of HPT patients, we minimized the risk of selection bias towards patients with more severe HPT. Moreover, we compared parameters of renal function between HPT patients and well-matched individuals from the general population. With 21%, we observed a higher prevalence of renal insufficiency in our HPT patient cohort. A high proportion of HPT patients (41%) presented hypercalciuria. Renal calcifications occurred in 10%.

The observed prevalence of renal insufficiency is in accordance with previous prospective studies reporting prevalences ranging from 12% to 23% (10, 12, 14). However, these figures are much lower than the results of a retrospective study including 120 HPT patients describing renal insufficiency in 41% of HPT patients (9). This difference may be due to a selection bias towards patients with more severe HPT possibly overestimating the prevalence in the retrospective analysis. Nevertheless, the prevalence of renal insufficiency remains increased in HPT patients compared to matched individuals, highlighting the importance for regular monitoring of renal function and control of modifiable additional risk factors as diabetes mellitus and hypertension.

Risk factors for an impaired renal function among HPT patients remain unclear. Not unexpectedly and comparable to previously published results, eGFR inversely correlated with age as well as disease duration in our analysis (9, 11, 14, 16). Moreover, we found a slightly but significantly elevated diastolic blood pressure despite more frequent use of several groups of antihypertensive drugs in HPT patients. Interestingly, a high calcium excretion was associated with a higher eGFR in our and previously published studies (42, 43). This might be due to a higher glomerular filter calcium load in combination with a higher eGFR (44). In our study, HPT patients presented higher phosphate and lower magnesium serum levels. Hyperphosphatemia might increase the risk for renal insufficiency by disrupting endothelial function of the glomerular filtration barrier (45). Hypomagnesemia may serve as additional risk factor for renal insufficiency through endothelial dysfunction, inflammation and vascular calcification (46). The role of PTH in relation to renal insufficiency in HPT remains unclear. PTH binds to PTH1-receptor on glomerular endothelial cells and proximal tubular cells inducing calcium reabsorption and phosphate excretion (47, 48). Some studies showed a significant decline in eGFR after parathyroidectomy (49, 50), whereas others showed a beneficial effect of parathyroidectomy on renal function (48, 51, 52).

Our data revealed no association of renal calcifications with renal function impairment. Other studies examining HPT showed an association between renal insufficiency and serum albumin-corrected calcium (16, 24, 25) or calcium-phosphate product (16, 53) which was not the case in our patient series.

Our study presents a comparatively large series of 150 systematically performed renal ultrasounds in HPT patients (5, 10, 12, 14, 19). We found renal calcifications in 10.0% of patients divided in 3.3% nephrolithiasis and 7.3% nephrocalcinosis (one patient had both nephrolithiasis and nephrocalcinosis). Saha et al. reported a similar prevalence for renal calcifications (11%), nephrolithiasis (5%) and nephrocalcinosis (6.7%) in their cohort of non-surgical HPT (14). In some retrospective studies a higher prevalence of renal calcifications ranging from 31% to 51% has been reported (9, 13, 54). In these studies, ultrasound examinations were only performed in 33% to 48% of the included patients, leading to a possible selection bias as more patients with suspected kidney disease may have been investigated. The reported prevalence of nephrolithiasis in the general population is 4.7% in Germany and 8.4% in the United States (55, 56). Based on these data, there was no evidence of an increased prevalence of nephrolithiasis in our patient cohort. However, we might have over- or underestimate patients with this condition due to the inherent limitation of ultrasound as a detection tool for renal calcifications especially in comparison to computed tomography. In contrast to our observations, some (10, 19, 57) but not all other studies (14) found an increased risk for nephrolithiasis among HPT. With regard to nephrocalcinosis, the risk for HPT patients was consistently found to be increased (14, 57).

Predictors and risk factors leading to renal calcifications in HPT remain largely unclear. In our study, patients with renal calcifications had a higher urinary calcium excretion and calcium-to-creatinine ratio. The daily dose of diuretics was higher in patients with renal calcifications which was most likely a consequence of observed renal calcifications or hypercalciuria. Similar to our findings, a higher 24-hour calcium excretion in patients with renal calcifications was found in four studies (12, 14, 17, 40). Thus, hypercalciuria seems to be a relevant factor for renal calcification. In general, a high prevalence of hypercalciuria was found in 41% of HPT patients corresponding to previously reported prevalences of 26% to 54% in smaller prospective studies (10, 12, 19, 31). A single morning fasting calcium value is not fully reflective of calcium levels throughout the day, and the clinical symptoms can vary significantly between individuals (58). Therefore, in the presence of hypercalciuria, even with normal findings for serum calcium, over-replacement should be considered. However, only 10 patients presented with normocalcemia, hyperphosphatemia and hypercalciura in our cohort. The link between hypercalciuria and serum albumin-corrected calcium underlines the importance of careful monitoring of this electrolyte. Despite established therapy including thiazides, hypercalciuria persisted in a high number of HPT patients. Interestingly, 51% of HPT patients with concomitant thiazide treatment still presented with hypercalciuria raising the question, whether thiazides sufficiently address hypercalciuria.

In our study, logistic regression analysis revealed an association between disease duration and serum phosphate or disease duration and 24-hour urinary calcium excretion with renal calcifications, depending on exclusion of patients with rhPTH therapy. In contrast to a previous study from India (14) and a study including 29 children with HPT (24), a higher calcium-phosphate-product could not be found to be a significant predictor for nephrocalcinosis in the present study. But again, both Saha et al. and Levy et al. almost exclusively examined patients with non-surgical HPT. None of our patients had a calcium-phosphate-product above the upper limit of 4.4 mmol^2^/L^2^ indicating that this threshold is less helpful for monitoring patients in order to prevent renal calcifications.

There are some strengths as well as limitations to our results. The relatively small number of 12 patients with detected renal calcifications made it difficult to accurately assess any risk factors. Identification of risk factors for renal complications necessitates a longitudinal prospective study. We contacted all patients diagnosed with “hypoparathyroidism” in the medical reports of our hospital since 2014. The voluntary nature of participation and the setting at a tertiary care hospital still makes a certain selection bias likely. Nevertheless, we recruited a comparably large cohort, including patients new to our university hospital. Questionnaires, devices and observers differed between our study and the population-based SHIP study providing matched individuals and systematic differences cannot be completely ruled out. However, assessment of both cohorts was conducted according to highly standardized protocols by qualified personnel. Moreover, the same equations or definitions were applied in both cohorts to define the endpoints of interest.

This study is a large-scale, cross-sectional, real-life study carefully assessing renal complications in HPT patients compared to well-matched individuals. One further key strength of our study is the performed renal ultrasound in 93% of our study population minimizing a potential selection bias towards patients with more severe HPT. Another strength of our study is the comparison of our patients to well-matched individuals considering the impact of age group, sex, BMI, smoking, diabetes mellitus and hypertension.

## Conclusion

In this study we found an increased prevalence of impaired renal function among patients with chronic HPT compared to matched individuals of the general population though it was not as high as described in retrospective studies previously. Furthermore, we found a high prevalence of hypercalciuria both in patients with and without thiazide treatment. The prevalence of renal calcifications was comparable to previously published prospective studies. In logistic regression model, disease duration, higher 24-hour urine calcium excretion and serum phosphate were associated with renal calcifications. In contrast to other studies, calcium-phosphate-product did not serve as a predictor for renal calcifications. In a clinical setting, we recommend maintaining serum phosphate and calcium excretion within reference ranges. Renal function should be monitored regularly. Hypercalciuria as an important risk factor for the development of renal calcifications often occurs under standard therapy with calcium and vitamin D. In this context, the treatment with parathyroid hormone replacing the physiological hormone function and thereby avoiding hypercalciuria appears to get more important. In addition, other risk factors leading to impairment of renal function, such as hypertension and diabetes mellitus, should be considered with greater attention and optimized if necessary.

## Data availability

The dataset generated during and/or analyzed during the present study are not publicly available but are available from the corresponding author on reasonable request.

SHIP-TREND data are not publicly available. The informed consent obtained from the participants of the SHIP studies does not cover data storage in public databases due to confidentially reasons. Data usage for scientific and quality control purposes can be applied for following a standardized procedure: http://www2.medizin.uni-greifswald.de/cm/fv/ship/daten-beantragen/. The corresponding author will, on request, detail the restrictions and any conditions under which access to data can be granted.

## Significance statement

This study is to our knowledge the largest prospective investigation on renal function and calcifications among patients with HPT. HPT patients present a higher prevalence of impaired renal function and hypercalciuria. However, the prevalence of renal calcifications was not as high as reported in retrospective studies. We observed a higher 24-hour urine calcium excretion and lower serum magnesium as main predictors. As hypercalciuria, one main risk factor of renal calcifications, often occurs under standard therapy with calcium and vitamin D, treatment with parathyroid hormone is getting more important as it allows to avoid hypercalciuria. Moreover, calcium-phosphate-product with the currently used reference ranges does not seem to be a valuable parameter for treatment monitoring in order to prevent renal calcifications.

## Data availability statement

The raw data supporting the conclusions of this article will be made available by the authors, without undue reservation.

## Ethics statement

The studies involving humans were approved by Ethics Committee of the University of Würzburg. The studies were conducted in accordance with the local legislation and institutional requirements. The participants provided their written informed consent to participate in this study.

## Author contributions

Conceptualization: CTF, SH. Design of study, study protocol, applications for authorities: CTF, SH. Drafting manuscript: KG, CTF. Review manuscript: CTF, AH, HV, SH. Data analysis: KG, AH, CTF. Conduct of study: KG, CTF, FH, AP, A-CK, HV, SH. All authors contributed to the article and approved the submitted version.

## Supplementary material

Supplementary Table 1 Characteristics of HPT patients and healthy individuals after 1:3 matching for age group (20-39, 40-49, 50-59, ≥60 years), sex, BMI ( ± 3 kg/m²), current smoking, diabetes mellitus, hypertension and intake of thiazides.

Supplementary Table 2 Proportion of HPT patients and matched SHIP-TREND individuals with eGFR<60ml/min/1.73m² stratified by age group (matching for age group (20-39, 40-49, 50-59, ≥60 years), sex, BMI ( ± 3 kg/m²), current smoking, diabetes mellitus, hypertension and intake of thiazides).

Supplementary Table 3 Binary logistic regression analysis with the endpoint renal calcifications and the independent variables 24-hour urine calcium excretion, serum phosphate, serum magnesium, serum calcium-phosphate-product and disease duration. CI, confidence interval.

Supplementary Table 4 Binary logistic regression analysis with the endpoint renal calcifications and the independent variables 24-hour urine calcium excretion, serum phosphate, serum magnesium, serum calcium-phosphate-product and disease duration after the exclusion of all patients with HPT receiving rhPTH replacement therapy. CI, confidence interval.
