# Supplementary material for: Renal complications in chronic hypoparathyroidism – a systematic cross-sectional assessment
Source: Front Endocrinol (Lausanne). 2023 Nov 2;14:1244647. doi: 10.3389/fendo.2023.1244647 (PMC10654620; doi:10.3389/fendo.2023.1244647)
Supplement: Supplementary Table 1 — Characteristics of HPT patients and healthy individuals after 1:3 matching for age group (20-39, 40-49, 50-59, ≥60 years), sex, BMI ( ± 3 kg/m²), current smoking, diabetes mellitus, hypertension and intake of thiazides. [file DataSheet_1.docx]

Supplementary Material

**Renal Complications in Chronic Hypoparathyroidism – a Systematic Cross-Sectional Assessment**

**Gronemeyer K^1*^, Fuss CT^1*^, Hermes F^1^, Plass A^1^, Koschker AC^1^, Hannemann A^2,3^, Völzke H^3,4^, Hahner S^1^**

**Correspondence:** Stefanie Hahner, [Hahner_S@ukw.de](mailto:Hahner_S@ukw.de)

**Supplementary Tables**

**Table S1:** Characteristics of HPT patients and healthy individuals after 1:3 matching for age group (20-39, 40-49, 50-59, ≥60 years), sex, BMI (±3 kg/m²), current smoking, diabetes mellitus, hypertension and intake of thiazides.

| **Characteristics** | **HPT patients** | **SHIP-TREND** n=366 | **p-value** |
| --- | --- | --- | --- |
|  | n=122 |  |  |
| Female, % | 75.4 | 75.4 | - |
| Age, years | 57.0 (48.0 – 64.0) | 56.0 (46.0 – 65.0) | 0.74 |
| BMI, kg/m² | 26.6 (23.6 – 30.5) | 26.6 (23.9 – 31.0) | 0.81 |
| Current smoking, % | 13.1 | 13.1 | - |
| Diabetes mellitus, % | 2.46 | 2.46 | - |
| TSH, mU/L | 0.65 (0.10 – 1.60) | 1.16 (0.74 – 1.58) | <0.01 |
| Serum total calcium, mmol/L† | 2.10 (2.00 – 2.30) | 2.29 (2.23 – 2.34) | <0.01 |
| Serum albumin-corrected calcium, mmol/L† | 2.03 (1.88 – 2.12) | 2.31 (2.24 – 2.36) | <0.01 |
| Serum magnesium, mmol/L† | 0.79 (0.73 – 0.83) | 0.86 (0.81 – 0.91) | <0.01 |
| Serum phosphate, mmol/L†^,^** | 1.32 (1.17 – 1.45) | 0.99 (0.88 – 1.09) | <0.01 |
| Systolic BP, mmHg* | 127 (118 – 138) | 127 (116 – 140) | 0.92 |
| Diastolic BP, mmHg* | 81.5 (75.0 – 90.0) | 77.0 (70.0 – 83.5) | <0.01 |
| Hypertension, % | 56.6 | 56.6 | - |
| Intake of |  |  |  |
| any antihypertensive medication, % | 43.4 | 45.1 | 0.62 |
| diuretics, % | 12.3 | 10.4 | 0.27 |
| betablockers, % | 22.1 | 27.9 | 0.15 |
| calcium channel blockers, % | 11.5 | 6.28 | 0.05 |
| RAAS Inhibitor, % | 31.2 | 32.2 | 0.77 |
| other antihypertensives, % | 0.00 | 1.09 | 0.25 |
| eGFR, ml/min/1.73m² | 74.4 (64.4 – 85.1) | 96.1 (83.7 – 103.5) | <0.01 |
| Urine albumin-to-creatinine, mg/g | 8.93 (6.17 – 12.32) | 11.01 (6.75 – 18.88) | <0.01 |
| Albuminuria, % |  |  | 0.23 |
| None, % | 92.6 | 88.8 |  |
| Microalbuminuria, % | 6.56 | 10.4 |  |
| Macroalbuminuria, % | 0.82 | 0.82 |  |

| *1 missing value in HPT patients and SHIP-TREND participants, respectively |
| --- |
| **1 missing values in SHIP-TREND participants |
| † Normal values: serum total calcium 2.0 – 2.7 mmol/L, serum albumin-corrected calcium 2.0 – 2.7 mmol/L, serum phosphate 0.87 – 1.45 mmol/L, serum magnesium 0.70 – 1.05 mmol/L |

**Table S2:** Proportion of HPT patients and matched SHIP-TREND individuals with eGFR<60ml/min/1.73m² stratified by age group (matching for age group (20-39, 40-49, 50-59, ≥60 years), sex, BMI (±3 kg/m²), current smoking, diabetes mellitus, hypertension and intake of thiazides).

| Age group | eGFR<60, in % | | p |
| --- | --- | --- | --- |
|  | HPT patients | SHIP-TREND |  |
|  | (n=122) | (n=366) |  |
| 20-39 | 0.00 | 0.00 | - |
| 40-49 | 4.76 | 0.00 | 0.08 |
| 50-59 | 12.2 | 0.00 | <0.01 |
| ≥60 | 40.0 | 3.70 | <0.01 |
| total | 19.7 | 1.40 | <0.01 |

**Table S3:** Binary logistic regression analysis with the endpoint renal calcifications and the independent variables 24-hour urine calcium excretion, serum phosphate, serum magnesium, serum calcium-phosphate-product and disease duration. CI, confidence interval.

|  | Odds Ratio | 95% CI for Odds Ratio | p-value |
| --- | --- | --- | --- |
| 24-hour urine calcium excretion, mmol/24h | 1.137 | 0.999 – 1.294 | 0.053 |
| Serum phosphate, mmol/dL | 0.733 | 0.563 – 0.953 | 0.020 |
| Disease duration, years | 1.063 | 1.021 – 1.106 | 0.003 |

**Table S4:** Binary logistic regression analysis with the endpoint renal calcifications and the independent variables 24-hour urine calcium excretion, serum phosphate, serum magnesium, serum calcium-phosphate-product and disease duration after the exclusion of all patients with HPT receiving rhPTH replacement therapy. CI, confidence interval.

|  | Odds Ratio | 95% CI for Odds Ratio | p-value |
| --- | --- | --- | --- |
| 24-hour urine calcium excretion, mmol/24h | 1.215 | 1.058 – 1.396 | 0.006 |
| Serum magnesium, mmol/dL | 0.436 | 0.182 – 1.049 | 0.064 |
| Disease duration, years | 1.042 | 0.998 – 1.088 | 0.063 |
